# A practical review of magnetic resonance imaging for the evaluation and management of cervical cancer

**DOI:** 10.1186/s13014-016-0591-0

**Published:** 2016-02-02

**Authors:** Emma C. Fields, Elisabeth Weiss

**Affiliations:** Virginia Commonwealth University, Richmond, VA USA

**Keywords:** Cervical cancer, Magnetic resonance imaging, Radiotherapy

## Abstract

Cervical cancer is a leading cause of mortality in women worldwide. Staging and management of cervical cancer has for many years been based on clinical exam and basic imaging such as intravenous pyelogram and x-ray. Unfortunately, despite advances in radiotherapy and the inclusion of chemotherapy in the standard plan for locally advanced disease, local control has been unsatisfactory. This situation has changed only recently with the increasing implementation of magnetic resonance image (MRI)-guided brachytherapy. The purpose of this article is therefore to provide an overview of the benefits of MRI in the evaluation and management of cervical cancer for both external beam radiotherapy and brachytherapy and to provide a practical approach if access to MRI is limited.

## Introduction

Cervical cancer is a leading cause of mortality in women worldwide [1]. The primary treatment of very early stage disease (IA1-IB1) is surgery and for more advanced disease (larger IB1-IVA) radiotherapy combined with chemotherapy [2–6]. Clinical staging based on physical exam combined with multimodality imaging is of utmost importance as it determines whether a woman is eligible for surgery and also describes the extent of the cancer for women who are not surgical candidates.

Definitive radiation therapy of cervical cancer is one of the most challenging treatment situations in radiation oncology requiring application of high radiation doses to achieve tumor control. However, the geometrical distribution of target volumes in the pelvis with a central tumor positioned directly between radiosensitive organs (bladder, rectum, sigmoid and small bowel) and involved or at risk lymph nodes neighboring small bowel are challenging for external beam therapy. High radiation doses can therefore typically not be delivered by external beam radiotherapy alone and necessitate an additional brachytherapy boost.

Traditionally, the brachytherapy boost has been delivered with tandem-based applicators using 2D x-ray planning with a pear-shaped dose distribution prescribed to Point A. However, comparisons of point-based 2D planning with volumetric-based 3D planning show poor correlation between point doses and both coverage of clinical target volumes as well as doses to organs at risk (OARs), indicating a clear need for improved image guidance and soft tissue-based information to guide the planning process [7].

With its excellent soft tissue imaging characteristics, magnetic resonance imaging (MRI) now plays an important role in many aspects of tumor staging, planning and delivery of radiotherapy, post treatment response assessment and surveillance [8–10]. MRI is well known to have superior soft tissue imaging characteristics and shows excellent results in determining local disease extent compared to physical exam and other 3D imaging techniques [11, 12]. For women who are managed with primary radiotherapy, MRI also provides clear visualization of the cervical tumor in multiple planes allowing for a reliable volumetric definition of the target volume both for external beam and brachytherapy treatment planning [9]. Particularly, with the addition of MRI to brachytherapy planning, tumor control rates are significantly improved [13–15]. Additionally, MRI is very useful in determining response to treatment after a course of chemoradiation, as well as determining late effects in the normal tissues [10, 16, 17].

Logistically, it is often difficult to obtain MRIs on cervical cancer patients at multiple time points during treatment. However, there are workarounds that are practically useful for centers without dedicated MR scanners in their department. There are promising new directions in the realm of MR imaging with increased use of morphological and functional MRI for adaptive radiotherapy and promising results showing better evaluation of treatment response and improved outcomes for patients [18–21]. The purpose of this article is to review the benefit of MRI for radiotherapy of cervical cancer and provide recommendations for use of MRI that are applicable to daily practice.

### MRI for pre-treatment assessment of cervical cancer

The International staging system for cervical cancer is based on clinical exam, as the primary management is typically non-surgical. The only modalities included in the International Federation of Gynecology and Obstetrics (FIGO) staging system are physical exam, cystoscopy, proctoscopy, intravenous urography and x-rays. However, where imaging resources are more widely available MRI, computed tomography (CT) and positron emission tomography (PET/CT) are used for their anatomical detail, to guide treatment planning and provide prognostic information.

MRI has become a key component of initial staging for cervical cancer due to the superior soft tissue delineation of pelvic structures. For women with early-stage cervical cancers, MRI is helpful for determining the appropriateness of surgery verses primary chemoradiation. In fact, several studies using pre-operative MR imaging have demonstrated that in patients with early-stage invasive cervical tumors (<3 cm, confined to the cervix) MRI has a 94 % accuracy and 95 % negative predictive value for determining parametrial invasion at the time of diagnosis. Using MRI with fast spin-echo (FSE) T2-weighted technique it was possible to determine who should be managed surgically and who should be managed with chemoradiation in order to try to avoid tri-modality therapy with its known complications [22–25].

For women who were planned for curative surgery, the prospective ACRIN 6651/GOG 183 Intergroup study used MRI and CT pre-operatively and showed that in patients with stage ≥ IIB MRI had the highest sensitivity for disease detection (53 %) compared to CT (42 %) and FIGO clinical stage (29 %) [12]. From the same group of patients, MRI was found to be better than CT and clinical exam for evaluating uterine body involvement and measuring tumor size, but neither were good for stromal invasion. In a retrospective analysis of the same study, interobserver variability was assessed between CT and MRI for diagnostic accuracy. MRI showed higher inter-rater agreement as well as better tumor visualization and detection of parametrial invasion [11].

For women with larger primary tumors where chemoradiation is the preferred definitive treatment, MRI is helpful in determining pelvic extent of disease in order to plan for radiation treatments and may even obviate the need for more invasive procedures such as exam under anesthesia [26, 27]. Recent guidelines for staging of cervical cancer using MRI published by the European Society of Urogenital Radiology recommend MRI for staging tumors that are clinically at least IB1 and also for smaller tumors if fertility preserving surgery is a consideration. They recommend getting images in at least 2 planes (sagittal, coronal, axial) with T2 weighted sequences as this best detects extension into uterus, parametria and adjacent organs (Table 1) [8].

**Table 1:** Recommended MR imaging

MR study	Plane orientation	EBRT	BT	Follow up
T2 FSE		Recommended	Recommended	Recommended
	• Axial			
	• Para-axial (perpendicular to the long axis of the cervical canal)	GTV (bright) Delineate OARs Use for extension into uterus, parametria and adjacent organs	GTV (bright)	Exclude residual high signal intensity in cervix High signal in OARs can reflect inflammation Low signal in OARs can reflect fibrosis
	• Para-coronal (parallel to the long axis of the cervical canal)			
	• Sagittal			
T1 weighted		Optional Delineate OARs	Optional	Optional
DW-MRI with ADC		Optional GTV (dark)	Optional	Optional ADC increases with treatment response

In nearly all cases, MRI provides additional information to standard clinical exam, CT and PET/CT imaging as it allows improved assessment of the extent of primary tumor disease, particularly to determine tumor diameter and infiltration of the parametria. This information is important to denote prior to embarking on definitive management, either surgically or with chemoradiation.

### MRI for external beam radiotherapy planning

The most clinically relevant benefit of MRI for cervical cancer planning lies in the clear visualization of the cervical tumor in multiple planes allowing for a reliable volumetric definition of the target volume.

Traditionally, the pelvic treatment fields for cervical cancer were designed based on bony landmarks using 2D planning. However, early studies with MR imaging assessed tumor coverage with standard pelvic fields, and it was observed that aligning to bony anatomy resulted in tumor miss in up to 66 % [28–31]. As treatments are becoming more conformal, MRI is needed to ensure that the target is adequately covered.

For treatment planning, MRI should be performed in the same position as the planned treatment, either using an MR simulation or with co-registration of images to a CT simulation. Images should be obtained with 1.5-3 T scanners with body coils. To control for peristaltic motion 0.5 mg glucagon IV can be given prior to and halfway through the exam. In general, multiplanar T2 weighted images are most helpful for defining both the target and OARs (Table 1) [9].

Recently, there have been contouring guidelines issued for external beam planning of cervical cancer by both the Gynecologic IMRT Consortium and the Japanese Radiation Oncology Group (JCOG). These guidelines each recommend delineation for both target and OARs on T2 weighted MRI and demonstrated that compared with contouring on CT, contouring on MRI showed improved reproducibility among experts, particularly in the region of the parametrium. Both groups acknowledged that most physicians use primarily CT-based planning, but the consensus was that for conformal planning T2-weighted MRI was strongly recommended [32, 33]. Based on these findings, MRI in treatment position has become a standard imaging procedure for 3D conformal and intensity modulated external beam treatment planning [34].

Another factor that strongly influences external beam treatment precision is the large inter- and intrafraction mobility of pelvic organs that affect both target volume dose coverage and dose to sensitive OARs. MRI is particularly useful to measure motion variability due to its excellent soft tissue visualization, the absence of radiation, the availability of multiplanar imaging and fast 4D imaging. Using serial MRIs during external beam radiation for cervical cancer demonstrates that the uterus, cervix, vagina and even pelvic lymph nodes have considerable motion between treatments due to changes in bladder filling, rectal filling and other internal motion. Uterine motion is the most significant of these, with average interfraction motion of 7 mm in the superior to inferior and anterior to posterior directions [35]. Intrafraction motion has also been shown of up to 10 mm for the CTV when using sequential MRI for patients, even when done within 16 min of each other [36]. However, to compensate for the observed organ motion, large CTV and PTV margins are needed and result in inclusion of large volumes of normal tissue in the target volume causing potentially detrimental side effects that reduce the overall therapeutic ratio. Various solutions have been suggested, e.g. anisotropic and tapered population-based margins with larger margins at the fundus compared to the cervix, individualized margins based on the observation of large interpatient variations [37, 38]. A more practical solution is to incorporate intrafraction and interfraction MRI-guided soft tissue registration with adaptation of the treatment plan to adjust for the observed variations [36].

### MRI for brachytherapy planning

While MRI for external beam treatment planning is still in development, over the last years the availability of MRI has transformed brachytherapy for cervical cancer.

With improved visualization of the residual tumor extent at the time of brachytherapy, new applicators with interstitial needles have been added to the existing repertoire of applicators to cover particularly lateral extensions of tumor into the parametria that would have been under dosed with classical applicator configurations [39]. In addition, a new MRI-based nomenclature of target volumes has been introduced in parallel with new guidelines for dose reporting created by The Groupe Européen de Curiethérapie and the European Society for Radiotherapy & Oncology (GEC-ESTRO). This working group was founded in 2000 to support, promote and standardize 3D based treatment planning for cervical cancer brachytherapy and have thus far 4 parts to the guidelines that focus on various aspects of MRI-based 3D brachytherapy. The new guidelines and MR imaging facilitate individualized treatment planning for brachytherapy, a process called MR image-guided adaptive brachytherapy [40–42].

MRI guided brachytherapy allows for a transition from prescribing uniformly to point A to targeting the residual disease in the cervix and paracervical tissues at the time of brachytherapy. The first part of the GEC-ESTRO working group recommendations created a common language for contour definition and prescription volumes at the time of brachytherapy. The GTV is defined by the clinical examination in addition to the high signal intensity regions in the cervix and paracervical region on FSE T2 weighted MRI. The GTV should be identified at diagnosis (GTV_D_) and at each brachytherapy insertion (GTV_B1_, GTV_B2_, etc.) to determine the tumor response and identify the regions at highest risk at the time of brachytherapy. The high risk CTV (HR CTV) is the region surrounding the GTV, including the whole cervix, with the highest risk of local recurrence due to residual macroscopic disease at the time of brachytherapy. The intermediate risk CTV (IR CTV) is a further extension of the HR CTV with a margin of 5-15 mm to encompass the region at risk for microscopic residual disease. The GEC-ESTRO recommendations are to prescribe to the HR CTV with no PTV margins. Ideally the IR CTV would receive at least 60Gy [41].

In the second working group guidelines, dose volume parameters are described for each of the target volumes described above as well as OARs. These parameters allow conversion from point-based prescriptions to 3D prescriptions and give recommendations for data collection for each brachytherapy application, including recording doses at point A as well as D100 and D90 (dose to 100 and 90 % of the volume) for GTV, HR CTV and IR CTV, respectively [42]. Additionally, instead of point doses for OARs, D_0.1cc_, D_1cc_, D_2cc_ are recorded and can be converted into the biologically equivalent dose in 2Gy fractions (EQD2) for addition to dose from external beam radiotherapy [43].

For these parameters to adequately be assessed and recorded, the MR imaging must be of a similar quality at each time point, using a 1.5-3 T magnet, surface pelvic coils, and patient preparation to minimize internal and external motion. For brachytherapy imaging with the applicator in place the recommended imaging includes T2 FSE in the para-axial, para-sagittal and para-coronal (according the cervix). In addition to the GTV, the OARs including the bladder, rectum, sigmoid and vagina are best contoured on the T2 weighted sequences [44]. For applicator reconstruction, para-transverse MR imaging is optimal with slice thickness ≤5 mm [45].

With the introduction of the GEC-ESTRO guidelines, many institutions have started using IGABT with MRI. Using these guidelines, with delineation of the GTV_BT_, HR CTV and IR CTV, due to more accurate assessment of residual disease at the time of the boost studies show considerable improvements in tumor control and reductions in normal tissue complications (Table 2). The Vienna group prospectively evaluated their results using MR IGABT during the years 2001–2008 and compared outcomes with CT assisted brachytherapy treatment planning during 1993–1997 and found an absolute local control benefit of 23–26 % and a relative reduction in local failure of 65 % [13]. These results have been replicated at other institutions, as shown in Table 2, with local control rates at 2 and 3 years of >90 %. Additionally, publications from Denmark and the Netherlands have shown that compared to conventional planning MR IGABT improves overall survival from 51-63 % with conventional planning to 79–86 % with the MR IGABT technique [14, 15]. With improved targeting, there has also been a reduction in treatment-related side effects with minimal severe late toxicity [13–15, 46–48].

**Table 2:** Studies using MR image-guided adaptive brachytherapy with outcomes

Study	Pts	Type of imaging for BT planning	Follow up (years)	LC (%)	DFS (%)	OS (%)
Simpson et al. (2015) [47]	76	CT 62 % had MRI with second fraction	2	94	70	75
Gill et al. (2015) [46]	128	MRI first fraction and then CT 51.6 % MRI 48.4 %	2	91.6	81.8	87.6
Rijkmans et al. (2014) [15]	83 43	MRI vs. x-ray	3	93 68		86 51
Lindegaard et al. (2013) [14]	140 99	MRI vs. x-ray	3	91		79 63
Nomden et al. (2013) [48]	46	MRI	3	93	71	65
Potter et al. (2011) [13]	156	MRI	3	95	75	68

*LC* Local control, *DFS* Disease free survival, *OS* Overall survival

A recent publication from the European study on MRI-guided brachytherapy in locally advanced cervical cancer (EMBRACE) describes women with FIGO IIB and IIIB disease as having either an infiltrative or expansive pattern of parametrial growth based on imaging characteristics. With repeat MR imaging at the time of brachytherapy, they found that women with infiltrative growth patterns had less response of their parametrial disease, but when using IGABT they were able to encompass gross disease into the HR CTV [49]. Further results from this large patient group are expected over the next several years (https://www.embracestudy.dk/).

With the ability to contour the target in 3D, it has become clear that previous fixed-geometry applicator based methods with point-based planning either delivered too large dose volumes for small and too small dose volumes for large tumors resulting in the risk of overdosing of normal tissues on the one side and under dosing the tumor on the other [7, 50, 51]. Even in small tumors, MRI-guided brachytherapy was found to reduce dose to the highest exposed 2 cc volumes (D2cc) to normal tissues by 12–32 % [52].

Despite the improved soft tissue visualization with MRI, many physicians are less familiar with the target delineation on MRI and recent comparison between CT and MRI contouring at the time of brachytherapy showed more agreement among experts with CT-based contours [53]. However, MRI was more accurate for patients with larger tumors, parametrial extension, or those with regression after external beam radiotherapy. Compared to MRI, CT has shown to produce large overestimation of residual disease at the time of brachytherapy and may therefore result in unnecessary dose to OARs [54].

### MRI for evaluation of treatment response

MRI has been applied clinically to determine the need for additional surgical salvage in the presence of residual tumor early after radiation therapy as well as to detect tumor recurrence during post treatment follow up. MRI has excellent accuracy in assessing residual tumor both for central recurrences in the vagina and cervix as well as tumor recurrences in the parametria and pelvic side walls that are difficult to detect with clinical exam [16]. In fact, the diagnostic ability to detect residual disease was found to be superior with MRI compared to PET with positive and negative predictive values for 74 % and 100 % for MRI compared with 44 % and 44 % for PET [17].

Similarly to other imaging modalities, the challenge with identifying tumor recurrence on MRI is differentiating between tumor and post therapy sequelae such as fibrosis, inflammation and necrosis. If obtained within 3–8 weeks post-treatment high false positive rates were observed, likely related to ongoing inflammatory changes [55]. A longer interval (>6 months) between treatment and post therapy assessment has shown improved reliability to correctly identify tumor recurrence [10].

### Logistical Issues with MRI

**Fig. 1:**
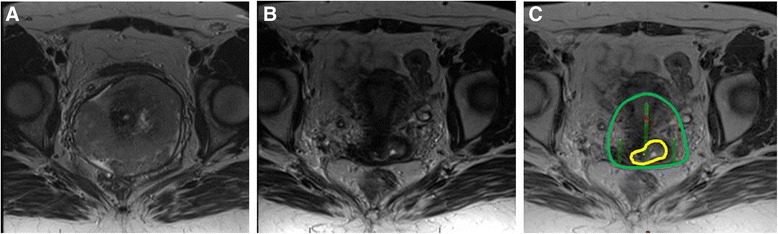
Axial T2 weighted MR images. **a**: Pre-treatment image with large cervical tumor. **b**: Pre-brachytherapy image showing excellent response to treatment with small focus of residual signal intensity. **c**: Simulated brachytherapy plan with GTV contoured (yellow) and plan adjusted to cover residual disease with 100 % isodose line (green) using tandem and ovoid applicators

**Fig. 2:**
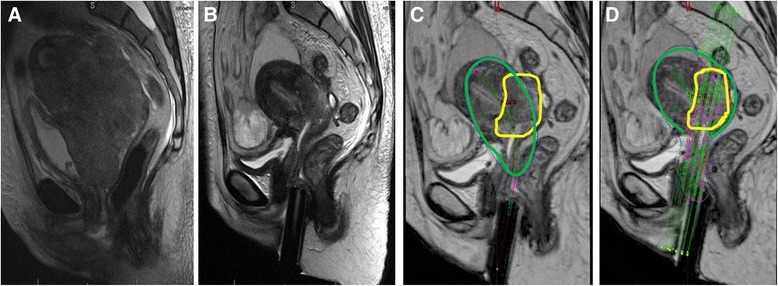
Sagittal T2 weighted MR images. **a**: Pre-treatment image with large cervical tumor. **b**: Pre-brachytherapy image with good response, but still large residual disease. **c**: Simulated brachytherapy plan with GTV contoured (yellow) and inadequate coverage with tandem and ovoids. **d**: Simulated plan with tandem and interstitial needles with excellent target coverage with 100 % isodose line (green)

One of the primary logistical issues with MRI for radiotherapy planning comes from limited access to MR scanners. While MRI simulation and real time MRI at the time of brachytherapy applicator insertion would be desirable for guidance, for many radiation oncology departments, MRI scans are obtained in the diagnostic radiology department for initial treatment planning and again after the applicator has already been inserted prior to the first brachytherapy session (Figs. 1 and 2) [9, 56].

**Fig. 3:**
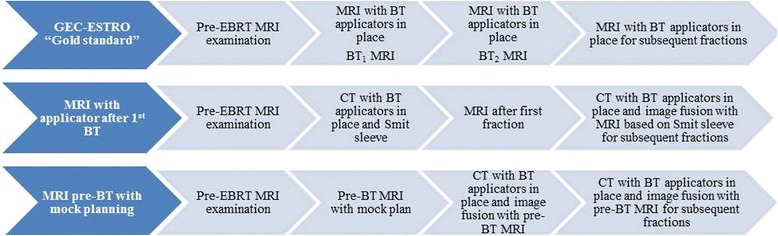
Work flow with incorporation of MRI with the GEC-ESTRO “gold standard” and 2 alternative approaches for limited MRI availability with MRI after 1^st^ BT and MRI pre-BT with mock planning

Ideally, MRI would be obtained with the applicator in place at each brachytherapy procedure. Several groups have recently published on workarounds for departments without dedicated MRI scanners (Fig. 3) [57, 58]. Performing both MRI and CT for the first brachytherapy fraction and then subsequent CT only imaging with registration to prior MRI is comparable to using MRI based planning for each fraction of brachytherapy. However, for larger tumors or for unfavorable geometry with organs at risk, MRI planning is still felt to be the gold standard [59, 60].

Despite of the benefits of MRI, there are some patients who are unable to have MR imaging. For example, patients with metallic implants, pacemakers, severe claustrophobia or a very large body habitus. In those patients, CT-based planning appears a reasonable alternative to MRI-based planning, as recently demonstrated in a large cohort of women treated with CT-guided brachytherapy planning with or without additional MRI. In this series of 76 women, 17 % had MRI prior to the first brachytherapy procedure without the applicator in place and 62 % had an MRI at the time of the second insertion with the applicator in place. Due to logistical issues with obtaining MRI, 21 % of patients were planned with CT alone. The 2 year local control was 94.2 %, disease free survival 75 % and overall survival 73 %, which is comparable to the results in the MR IGABT series in Table 2 [47].

Various challenges from medical physics perspective include the need for MR image registration with CT images for dose calculation, the effect of MR image distortion on geometrical accuracy, problems with intracavitary applicator visibility, susceptibility artifacts and reconstruction errors, and the need for MRI-compatible titanium or plastic applicators [39, 61].

Another limitation to the widespread use of MRI-based evaluation and treatment planning with MRI is physician discomfort with target and organ identification and target delineation in this platform. Many physicians are also less familiar with 3D treatment planning for brachytherapy and prefer using point-based planning. However, with the increased availability of clear guidelines in the literature as well as Continuing Medical Education (CME) opportunities to learn at the ABS GYN School and ASTRO contouring sessions, this will change over time [41, 42, 62–64].

### Future directions for the use of MRI in cervical cancer

Cervical cancer offers many opportunities for adaptive radiotherapy approaches with the goals of improving dose conformality, reducing normal tissue toxicity and potentially increasing tumor dose to areas of residual disease. These goals can be achieved by adjusting the dose plan based on individual patient characteristics of tumor volume regression, tumor motion and set up reproducibility. MRI plays an important role in the development of adaptive radiotherapy as it provides 3D information about organ motion and tumor volume shrinkage [35, 36, 65]. MRI-guided adaptive brachytherapy essentially has already incorporated the adaptive concept by adjusting the MRI-based brachytherapy dose plan of each fraction to the individual patient 3D imaging information obtained prior to each fraction [13, 15].

For external beam radiotherapy, no routine strategies of treatment adaptation have been established in clinic, in part due to challenges in deformable dose accumulation, the speed of adaptive re-planning, and limited resources. Inter- and intrafraction organ motion and tumor shrinkage offer various opportunities to adjust the individual plans to these parameters. Most of the tumor regression occurs during external beam radiotherapy with large interpatient variation in regression rates [66]. Depending on the timing of brachytherapy relative to external beam therapy, initial tumor volumes are reduced often by ≥80 % at the time of brachytherapy [16, 67–69]. Due to this large change in tumor volume, adaptive strategies are expected to increase the therapeutic ratio by enabling delivery of additional boost doses to residual tumor in the pelvic lymph nodes [70], residual central tumor [71, 72], or by selective sparing of high dose to organs at risk.

With MRI-linear accelerator development on the horizon, real-time MR imaging during treatment will allow for excellent soft tissue delineation, image fusion, rapid adaptive radiation planning, and improved tumor targeting for women with cervical cancer.

While morphological MRI has become a standard imaging method for cervix cancer radiotherapy, more recently, functional MRI sequences have been investigated as biomarkers for determination of radioresistance. Diffusion-weighted MRI (DW-MRI) is a non-contrast imaging methodology that measures water diffusion in tissue typically using apparent diffusion coefficients (ADC) for quantification and comparison. ADC values are lower in tumor than normal tissue due to higher cellularity and can subsequently increase as a result of therapy due to apoptosis and cell death, as well as inflammation and microvascular leakage [73–75]. Several small studies investigated DW-MRI in radiotherapy of cervical cancer and observed that ADC change early during radiotherapy predicts tumor response on MRI and is associated with overall survival [18–20]. It was also noticed that patients with high initial ADC values had worse outcomes than patients with lower ADC values, likely due to necrotic areas that were hypoxic and therefore radioresistant [76, 77]. Fluorodeoxyglucose (FDG) PET standard uptake value maximum and DW-MRI (ADC mininum) were found to be complementary prognostic factors for cervical cancer [78, 79]. However, DW-MRI is not routinely used in radiotherapy due to lack of larger clinical validation of results and lack of integration into the radiotherapy workflow [80].

Dynamic contrast-enhanced MRI (DCE MRI) is assumed to show vascular density and perfusion which is thought to be correlated with hypoxia and radioresistance. Changes in signal intensity indicating increasing tumor perfusion and volume change after 2–2.5 weeks of therapy predict local tumor control and survival [21]. In a large study with repeated DCE MRI before and during therapy, functional risk volumes were generated for subvolumes with critically low DCE signal. Larger functional risk volumes predicted tumor control and disease free survival before and during therapy. In fact, DCE MRI was a better predictor than anatomical volume change [81, 82].

Further new imaging methods under investigation include blood-oxygen level dependent (BOLD) MRI [83] and diffusion-weighted high-resolution magic angle spinning magnetic resonance spectroscopy (MRS) [84].

## Conclusions

Magnetic resonance imaging has been generating clinical interest for use in cervical cancer, but there has yet to be a comprehensive clinical review with practical information on the use of MRI for patients with cervical cancer. Using MRI, patients can be more accurately staged and guided towards the correct management options. For patients that require radiation therapy, MRI shows excellent soft tissue delineation and should be incorporated into both external beam and brachytherapy treatment planning. Ideally, availability of an MR scanner in the radiation oncology department is the most convenient, but even without dedicated scanners, obtaining MR imaging is practical and can be used for more accurate treatment planning and delivery. In the future, MRI will likely become even more prevalent as deformable dose registration, adaptive imaging for external beam and functional imaging become more established.

## Consent

Written informed consent was obtained from the patient for the publication of this report and any accompanying images.

## Abbreviations

ADC: apparent diffusion coefficients
BOLD: blood-oxygen level dependent
CT: computed tomography
DCE MRI: dynamic contrast-enhanced MRI
DFS: disease free survival
DW-MRI: diffusion-weighted MRI
EMBRACE: European study on MRI-guided brachytherapy in locally advanced cervical cancer
EQD2: biologically equivalent dose in 2Gy fractions
FDG: fluorodeoxyglucose
FIGO: International federation of gynecology and obstetrics
FSE: fast spin-echo
GEC-ESTRO: The Groupe Européen de Curiethérapie and the European society for radiotherapy & oncology
GTV_BT_: gross tumor volume at the time of brachytherapy
GTV_D_: gross tumor volume at the time of diagnosis
HR CTV: high risk clinical target volume
IGABT: image-guided adaptive brachytherapy
IMRT: intensity modulated radiation therapy
IR CTV: intermediate risk clinical target volume
IR CTV: intermediate risk clinical target volume
JCOG: Japanese radiation oncology group
LC: local control
MRI: magnetic resonance imaging
MRS: magnetic resonance spectroscopy
OARs: organs at risk
OS: overall survival
PET: positron emission tomography
T: Tesla
